# A New and Faster Test to Assess Vestibular Perception

**DOI:** 10.3389/fneur.2019.00707

**Published:** 2019-07-02

**Authors:** Bart Dupuits, Maksim Pleshkov, Florence Lucieer, Nils Guinand, Angelica Pérez Fornos, Jean Philippe Guyot, Herman Kingma, Raymond van de Berg

**Affiliations:** ^1^Faculty of Health, Medicine and Life Sciences, University of Maastricht, Maastricht, Netherlands; ^2^Division of Balance Disorders, Department of Otorhinolaryngology, Head and Neck Surgery, Maastricht University Medical Center, School for Mental Health and Neuroscience, Maastricht, Netherlands; ^3^Faculty of Physics, Tomsk State Research University, Tomsk, Russia; ^4^Service of Otorhinolaryngology Head and Neck Surgery, Department of Clinical Neurosciences, Geneva University Hospitals, Geneva, Switzerland

**Keywords:** vestibular perception, vestibular perceptual function, perceptual threshold, perceptual threshold measurement, vestibular function, vestibular function disorders

## Abstract

**Objective:** Clinical vestibular testing mainly consists of testing reflexes, but does not routinely include testing for perceptual symptoms. The objective of this study was to investigate a new and faster test for vestibular perception, and to compare its results with previous studies.

**Methods:** Fifty-five healthy subjects with no prior vestibular complaints were included and divided into three age groups. Vestibular perceptual thresholds were measured using a hydraulic platform in the dark. The platform delivered 12 different movements: six translations (forward, backward, right, left, up, and down) and six rotations/tilt (yaw left, yaw right, pitch forward, pitch backward, roll left, and roll right). The subject had to report the correct type and direction of movements. Thresholds were determined by a double confirmation of the lowest threshold. General trends in thresholds like relative interrelationship and the influence of age were analyzed and compared with values reported previously.

**Results:** Mean thresholds of age groups ranged between 0.092 and 0.221 m/s^2^ for translations, and between 0.188 and 2.255°/s^2^ for rotations. The absolute values differed from previous reports, but the relative interrelationship of thresholds between type and direction of motion remained. An association between age and vestibular thresholds was found, similar to previous reports.

**Conclusion:** This new and faster test for vestibular perception showed comparable patterns in perceptual thresholds when compared to more research oriented, lengthy tests. This might pave the way for establishing vestibular perception testing protocols useful for the clinic.

## Introduction

The vestibular organ consists of three semicircular canals (lateral, anterior, and posterior) and two otolith organs (saccule and utricle). Three major vestibular functions are gaze stabilization, spatial orientation, and balance. These essential functions also rely on the contribution of other multiple senses, such as the visual and somatosensory system (1). In case of vestibular failure, contributions of the visual and somatosensory system increase in order to maintain balance (sensory substitution). Concurrently, readjustments of brainstem vestibular processing and adaptation occur (2, 3).

Current diagnostics for the vestibular system mainly rely on the evaluation of reflexes, such as the vestibulo-ocular reflex (VOR) and the vestibulo-collic reflex. However, one third of patients with dizziness or imbalance have normal vestibular results on these tests (4). This illustrates that perceptual symptoms cannot always be addressed with current vestibular tests, and is probably related to the fact that vestibular perception utilizes other sensory pathways than vestibular reflexes (5). In general, perceptual thresholds have high sensitivity and specificity, since it is not easy to adapt to deficits caused by threshold-level stimuli. Therefore, there is a real clinical need to go “beyond reflexes” and measure vestibular perception, which could provide important additional information in the diagnostic process (4). Until now, vestibular thresholds have proven to be useful in identifying specific peripheral deficits and in diagnosing central disorders such as vestibular migraine (6–8). However, the clinical value of tests for vestibular perception is not yet fully determined. For example, they might develop into the equivalent of the “speech audiogram” for vestibular disorders (3, 6).

Vestibular perception has been tested previously with a platform capable of producing different motion profiles: yaw rotations, combined translational and rotational movements (5, 9), roll tilt (6), and lacked pitch movements (1, 8). The tested subject had to perceive and identify the type and/or direction of the movements. Next to this, differences between vestibular and visual thresholds were measured, and the effect of combining both was also evaluated (10, 11). However, these vestibular perception tests take considerable time: up to 3 h (6). This not only increases the burden for the patient, but might also decrease the attention of the patient during the test. These factors can significantly influence reliability and reproducibility of the results. Therefore, there is a need to develop a clinically oriented test for vestibular perception that is sensitive and specific, but less time-consuming.

The objective of this study was to investigate the application of a simplified and shorter paradigm for testing vestibular perception and to compare its results with those obtained in previous, research oriented studies. This new paradigm might be used in the future for multiple purposes, including clinical evaluation of the vestibular implant and diagnosis of vestibular perceptual deficits (4, 12).

It should be noted that vestibular perceptual tests are not purely testing the vestibular system (peripheral and central), since other sensory systems like proprioception are also involved in detecting movements. The brain integrates all these different inputs. Therefore, the vestibular perceptual thresholds can be considered as a functional outcome of the whole system, in which the vestibular system plays a major role (4).

## Methods

### Participants

Fifty-five healthy subjects with no prior vestibular complaints were included in this study. Ages ranged from 21 to 81 years old (median age 55 years, mean age 49 years). Twenty-four males and 31 females participated. Exclusion criteria comprised current vestibular disease, and inability to sit in the testing chair for at least 1 h. Patients with migraine or using vestibulosuppressants were also excluded because both these factors are known to influence vestibular function (7). All included subjects were able to complete the whole experiment.

### Perception Platform

Vestibular perceptual thresholds were measured using the hydraulic CAREN platform combined with the D-flow 3.22.0 software from Motek Medical BV (Amsterdam, The Netherlands). The platform delivered 12 different smooth, controlled movements: six translations (forward, backward, right, left, up, and down) and six rotations or tilt (yaw left, yaw right, pitch forward, pitch backward, roll left, and roll right). Each of the 12 thresholds was measured independently of others, which implies that no (major) effect should be expected from one movement on the thresholds of other movements.

### Preparations

The subject was informed about the testing paradigm. All subjects were tested by the same technician (BD). The subject was seated in a chair mounted on the platform, and then fastened with a seatbelt for security purposes and to limit information provided by the body sliding on the chair. The test was performed in complete darkness and a blindfold was put on to avoid any visual cues. An infrared camera was used to monitor the subject during the experiments. Subjects wore a headset for communication with the technician and to mask the surrounding noise of the platform by playing a mix of previous sound recordings of the platform. First, a practice run was performed to verify understanding of the testing paradigm and subject compliance. Then, the testing paradigm was carried out. The technician continuously checked and maintained attention of the patient by communicating via the headset.

### Testing Paradigm

The objective of the testing paradigm was to measure perceptual thresholds for angular and translational motions. Movements were applied in a random order and started at the highest possible accelerations. For each movement, the platform was first positioned and then the “test movement” was performed. After that, the platform returned to its neutral position. Then, the subject had to immediately report the direction and type of movement to the technician using the headset. Both the direction and type of movement had to be correct, in order for the response to be validated by the technician (i.e., to lower the acceleration for that specific movement). Translation accelerations were lowered in steps of 0.1 m/s^2^, rotation accelerations in steps of 10°/s^2^. In case of an incorrect or absent response, a step up of respectively, 0.05 m/s^2^ or 5°/s^2^ was used. If the response remained incorrect, the accelerations were increased again by 0.02 m/s^2^ and 2°/s^2^, respectively. The perceptual threshold for each movement was determined by a double confirmation of the lowest threshold, plus two times an absent response at the acceleration one step below the threshold.

### Stimulus

**Figure 1:**
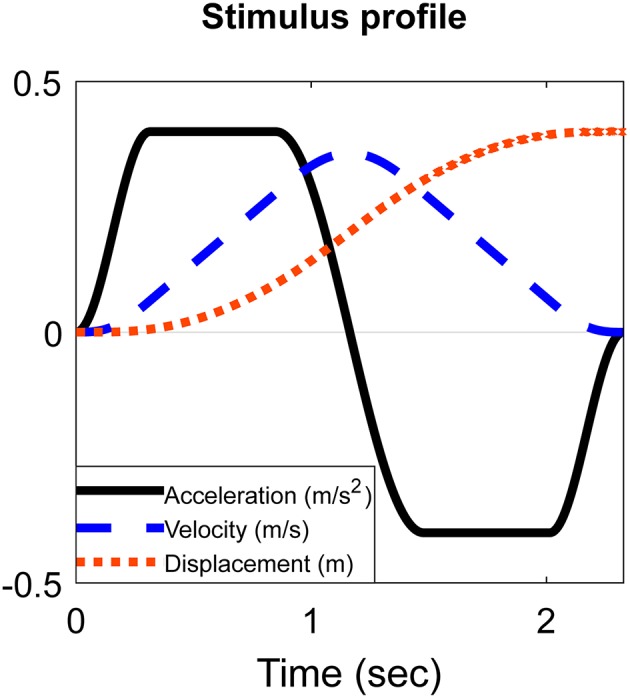
Schematic example of the stimulus shape of a translation.

A special motion stimulus profile was developed to quantify perceptual thresholds for translational (six directions) and rotational (six directions) accelerations. The motion profile for translational stimuli are illustrated in Figure 1. The rotational stimuli had the same profile. They were composed of a smoothly increasing acceleration phase (low jerk) until constant acceleration was obtained for a fixed duration (plateau phase). This was followed by a smooth decrease of the acceleration (low jerk) down to zero. After each stimulus the platform moved with a subthreshold acceleration and jerk to the starting position needed for the next chosen stimulus. By this procedure, patients did not feel any movement or tilt between the subsequent stimuli, by which it was not possible to anticipate on the type or direction of the next stimulus. A random sequence of all possible 12 stimuli was used. Due to the limitations of the platform, the range of translational movements was restricted up to 0.4 m, and the range of rotational movements up to 30°. This stimulus profile was chosen to provide a constant acceleration at a certain magnitude, for a given duration, defined by the investigator. All non-linear parts of the stimulus were sinusoidal to smoothly reach the plateau phases of the acceleration. The sine parameters (amplitude and frequency) depended on the magnitudes of acceleration (*a*) and jerk (*j*) and varied for each separate motion stimulus. Therefore, every stimulus was controlled by three parameters: maximum range, acceleration magnitude, and jerk magnitude. Minimum acceleration was 0.01 m/s^2^ for translations and 0.1°/s^2^ for rotations. Maximum acceleration was 0.4 m/s^2^ for translations and 40°/s^2^ for rotations.

### Data-Analysis

IBM SPSS Statistics Version 24 was used for data-analysis. In order to compare the type of movements, results were grouped into the same type of movement (e.g., translations and rotations), same type of translation (translation left and right, forward and backward, up and down), and same type of rotation (yaw, pitch forward and backward, roll left and right). To be able to statistically evaluate the influence of age, age groups were made for ages 21–39, 40–59, and 60–81 years. Mean thresholds of movements were calculated for the whole population of tested subjects, as well as for each age group separately. Paired *t*-tests were performed between all types of translations and between all types of rotations, to evaluate possible significant differences between them. Scatterplots were made for every movement tested by the platform to visualize the relation between perceptual thresholds and age. To further investigate the influence of age and gender, multiple regression analyses were performed for mean perceptual thresholds. The mean threshold of all movements, the mean threshold of all translations and the mean threshold of all rotations were used as dependent variables. Age and gender were used as independent variables. *P*-values below 0.05 were considered significant. Regarding the multiple regression analysis, Cooks distances were determined and a multicollinearity test was performed, showing no multicollinearity. In order to compare thresholds from previous literature (6) presented in velocity units (*v*), with the thresholds in this study presented in acceleration units (*a*), peak velocities were converted into peak accelerations by *a*_*peak*_ = *v*_*peak*_π*f*, where *f* was the motion frequency. Since both studies differed in terms of paradigm (determining thresholds differently, not all type of movements the same) and stimulus (different profile shape, duration, and frequencies), no statistics were applied to compare both datasets. However, general trends in thresholds like relative interrelationship and the influence of age were analyzed separately and compared between these studies.

### Ethical Considerations

The procedures in this investigation were in accordance with the legislation and ethical standards on human experimentation in the Netherlands and in accordance with the Declaration of Helsinki (amended version 2013). Approval was obtained from the ethical committee of Maastricht University Medical Center (NL52768.068.15/METC). All procedures were performed at the Maastricht University Medical Center. All subjects provided written informed consent.

## Results

### Perceptual Thresholds for Translations and Influence of Age and Gender

**Figure 2:**
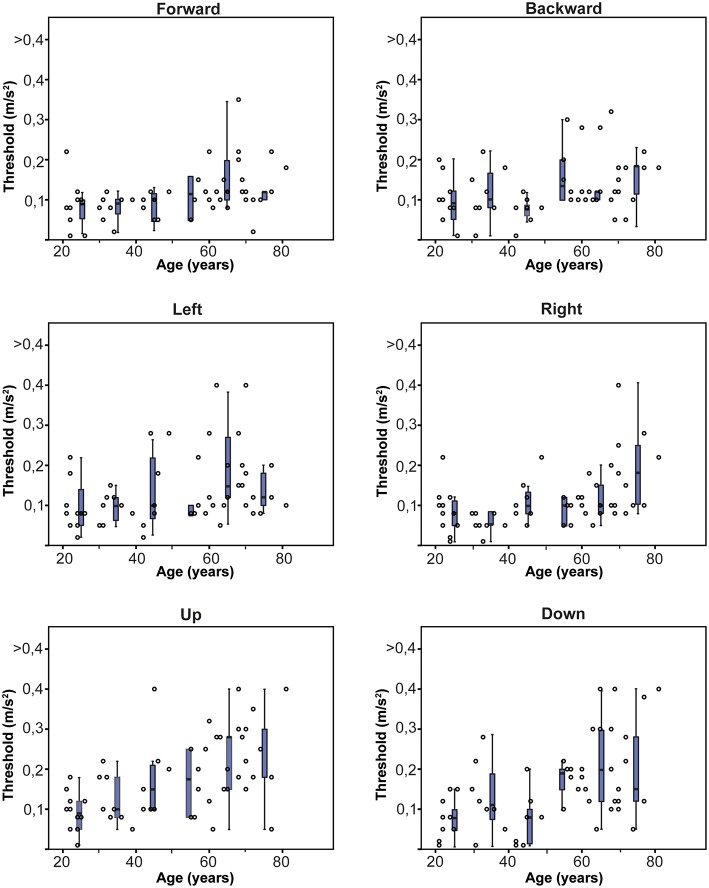
Normative thresholds for each direction of translation, obtained in 55 healthy subjects of different ages. Each dot represents the threshold of one subject for a specific translation. Each box plot represents the 25–75 percentiles of thresholds per decade, whiskers the 95 percentiles and bold black lines the median.

Thresholds for translations varied widely within and between age groups (Figures 2, **4**). Mean thresholds of age groups ranged between 0.092 and 0.221 m/s^2^ (Table 1). Thresholds of the upward-downward plane were significantly higher than those of the forward-backward plane (*p* = 0.03; Table 1). No significant differences were found between the other translations. Mean thresholds increased with age group, except for leftward and rightward translations. A multiple regression was run to predict the mean perceptual threshold of all translations from age and gender [*F*_(2, 52)_ = 12,480, *p* < 0.0005, *R*^2^ = 0.324]. Age added significantly to the prediction (*p* < 0.001), not gender (*p* = 0.240).

**Table 1:** Mean thresholds for translations and rotations, presented for each age group, with standard deviation between brackets.

**Age (in years)**	**No. of subjects**	**Translations forward + backward**	**Translations left + right**	**Translations up + down**	**Yaw rotations left and right**	**Pitches forward+ backward**	**Rolls left + right**
All	55	0.12 (0.05)	0.14 (0.11)	0.16 (0.09)	1.62 (1.59)	0.61 (0.89)	0.44 (0.85)
20–39	20	0.09 (0.04)	0.12 (0.15)	0.10 (0.05)	0.82 (0.56)	0.19 (0.24)	0.22 (0.55)
40–59	13	0.11 (0.04)	0.11 (0.06)	0.15 (0.07)	1.79 (1.67)	0.52 (0.52)	0.81 (1.42)
60–81	22	0.14 (0.05)	0.16 (0.08)	0.22 (0.09)	2.26 (1.89)	1.04 (1.20)	0.42 (0.55)

### Perceptual Thresholds for Rotations and Influence of Age and Gender

**Figure 3:**
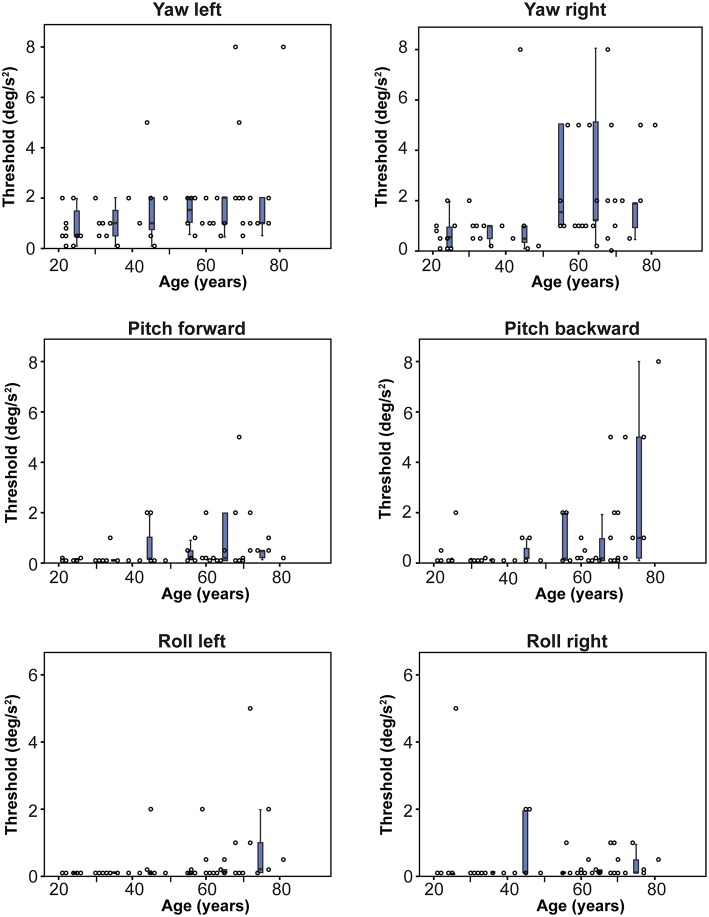
Normative thresholds for each direction of rotation, obtained in 55 healthy subjects of different ages. Each dot represents the threshold of one subject for a specific rotation. Each box plot represents the 25–75 percentiles of thresholds per decade, whiskers the 95 percentiles and bold black lines the median. Note that y-axes are optimized for each specific movement. Dots on the x-axis have a value of 0.01°/s^2^.

**Figure 4:**
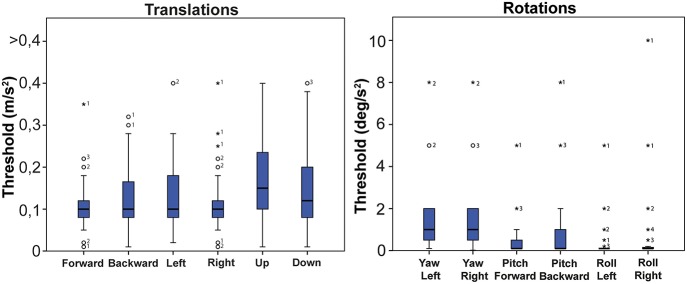
Normative values of all translations combined and all rotations combined. Bold black lines in the boxes represent medians, boxes the 25–75 percentiles, whiskers the 95 percentiles. Outliers are represented by an open circle, extreme outliers by an asterisk. Numbers next to a dot indicate the amount of dots with the same value.

Thresholds for rotations showed less variability within and between age groups than thresholds for translations (Figures 3, 4). Mean thresholds of age groups varied between 0.188 and 2.255°/s^2^ (Table 1). Perceptual thresholds for yaw rotations were significantly higher than for pitches and rolls (*p* = 0.016; Table 1). No significant difference was found between the pitches and rolls (*p* = 0.242). Mean thresholds increased with each age group for yaw and pitch rotations, but not for roll rotations. A multiple regression was run to predict the mean perceptual threshold of all rotations from age and gender [*F*_(2, 52)_ = 8,644, *p* < 0.005, *R*^2^ = 0.250]. Again, only age added significantly to the prediction (*p* < 0,001), not gender (*p* = 0.297).

### Comparison of Perceptual Thresholds With Previous Literature

**Figure 5:**
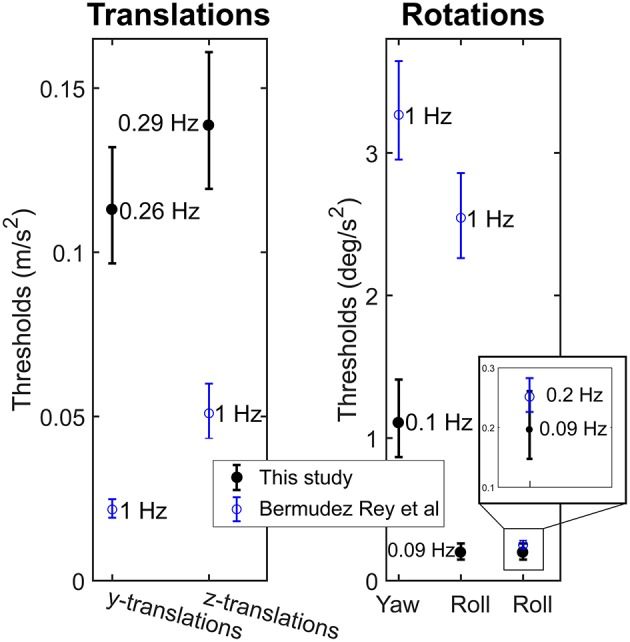
Mean and 95% confidence intervals of perceptual thresholds for translations and rotations in this study (*n* = 55), compared to those in previous literature (6) (*n* = 79). For each mean value, the frequency of the stimulus is given.

Figure 5 presents the perceptual thresholds for y- and z-translations and yaw and roll rotations in this study, compared to those in previous literature (6). Although the absolute thresholds varied between these studies, the relative interrelationship of thresholds between movements remained: y-translations and roll rotations showed lower mean thresholds than z-translations and yaw-rotations, respectively. Thresholds for roll rotations around 0.1 Hz in this study were close to those previously measured at 0.2 Hz. A significant age effect on thresholds was found in both studies (6).

## Discussion

This study was the first step in evaluating a clinically oriented test for vestibular perception. Perceptual thresholds in a group of healthy subjects were obtained and thresholds significantly increased with increasing age. Gender did not have a significant effect. These findings were congruent with a previous study, despite of this study using a different testing paradigm and different type of stimulus, and including more directions (6).

This testing paradigm differed from more research oriented studies in several ways. Firstly, it was devised to be relatively fast and complete, in order to have a test more suited for clinical settings. Testing time was substantially reduced from ~3 h to less than 1 h (45–60 min). This reduced burden for the patient, costs of testing, and might have improved attention of the patient. The latter is particularly relevant, since after a long testing session attention is more likely to decrease, resulting in less reproducible and reliable results (13). Testing time was reduced by using fewer motions to determine the thresholds. Reliability of thresholds was therefore ensured by adding pitches forward and backward to the types of movement, and by randomly presenting all stimuli in the same session, without the subject being aware of the type of movement. This reduced the possibility of reporting the right threshold by chance. Secondly, this testing paradigm used different stimuli than previously reported. It was based on a stimulus with the longest possible duration of constant peak acceleration (plateau phase) and varying frequencies, instead of a fixed frequency with a sinusoidally shaped acceleration profile. This new profile was chosen to have a longer exposition of the subject to the main parameter of the stimulus of interest and the main stimulus for the vestibular system: acceleration. However, due to the limitations of the platform, the frequency of the stimulus had to differ for each acceleration. This is a potential limitation, since frequency-dependency of the system is more difficult to evaluate. Next to this, it prevented comparison of the absolute thresholds of this study with previously reported ones. After all, the frequency-dependency of the vestibular system implies that testing at difference frequencies might yield different results (5, 6). Nevertheless, this could mainly explain the differences between the absolute values of thresholds between the studies. Thirdly, in this paradigm continuous interactive communication between the technician and the patient was added. In extensive preliminary trials, this strategy was found to be superior to using a joystick to indicate thresholds, without any significant communication. Communication also improved attention, reduced anxiety (since the patients sat in a dark room), and facilitated verification whether the reported thresholds were representative or not (e.g., a lack of attention at the moment of testing a certain threshold). If an unreliable threshold was suspected, the threshold was determined again. Fourthly, not all skin surfaces were covered to reduce somatosensory input as much as possible. Whether covering of all skin surfaces has any beneficial effects in this paradigm proposed, should still be determined.

More dispersion was observed in the thresholds for translations, than for rotations, pitches, and rolls. This was in accordance with previous literature (6) and could be attributed to a higher contribution of somatosensory input during these movements. Regarding the group of translations, thresholds of the vertical plane were significantly higher than those of the forward-backward plane. Regarding the group of rotations, thresholds for yaw rotations were significantly higher than those for pitches and rolls. It could be hypothesized that these two movements were less affected by somatosensory input, compared to the other movements in their group. For instance, a translation in the vertical plane will cause less activation of the somatosensory system, including neck proprioception (14), than translations in other planes, since the body remains in line with gravity. Also, a rotation in yaw plane does not include any tilt with respect to gravity, in contrast to pitches and rolls. These two movements appear therefore to be those that most purely test the thresholds for translations and rotations of the peripheral vestibular system, with the least interference of the somatosensory system.

The contribution of the somatosensory system implies that vestibular perceptual tests are not purely testing the vestibular system (peripheral and central), since somatosensory cues are also involved in detecting movements. The brain integrates all these different inputs. Therefore, the vestibular perceptual thresholds can be considered as a functional outcome of the whole system, in which the vestibular system plays a major role (4). This also implies that this test is not specifically designed to detect a peripheral or central vestibular deficit, but to demonstrate the vestibular perceptual functionality of a patient at a given time.

### Limitations

Many subjects could still hear some movements of the platform (e.g., translations downward) in spite of the masking noise on the headphones. Platform sounds were almost the same for each movement. Therefore, the sounds might have indicated that the platform was moving, but could not help in distinguishing between direction and type of movements (e.g., translations vs. rotations, upwards vs. downwards). Since the thresholds of movements were defined by the right type and direction of movements, it was hypothesized that sounds might have not significantly influenced the thresholds. However, the platform sounds should be taken into consideration when refining this testing paradigm.

Perceptual thresholds significantly increased with increasing age. Since the vestibular function of the healthy controls was not measured but only screened with a questionnaire, it cannot be determined whether the increasing thresholds with age were mainly influenced by age, or other factors. For example, age-related decline in vestibular function (presbyvestibulopathy) as well as clinically asymptomatic vestibulopathies could account for the decline of vestibular perception. This needs to be determined in future studies.

### Future

Next step is to investigate this testing paradigm in patients with unilateral and bilateral vestibulopathy. If this succeeds, it might pave the way for routinely measuring vestibular function “beyond reflexes.” It might be used in clinic, in which it should be noted that this test is relatively expensive regarding time and equipment, compared to other vestibular tests. Therefore, it is hypothesized that it will probably first be suited for tertiary referral centers that have the resources and interest to investigate vestibular perceptual threshold deficits in patients (regardless of the etiology), or to use it to demonstrate perceptual changes after rehabilitation. It could also be used in research settings to e.g., evaluate the effect on perceptual thresholds of future therapies, for example the vestibular implant (12, 13, 15–17). For the latter, it should be noted again that vestibular perception is the end-result of detection and processing of movements by the whole vestibular system (see above): peripheral and central. This process is susceptible to multisensory integration and many other factors (e.g., adaptation, compensation, and cognition) (18). Therefore, vestibular perception should be used in the future as an outcome measure by itself, and not purely as a marker of vestibulopathy.

## Conclusion

This new and faster test for vestibular perception showed comparable patterns in perceptual thresholds when compared to more research oriented, lengthy tests. This might pave the way for establishing vestibular perception testing protocols useful for the clinic.

## Ethics Statement

The procedures in this investigation were in accordance with the legislation and ethical standards on human experimentation in the Netherlands and in accordance with the Declaration of Helsinki (amended version 2013). Approval was obtained from the ethical committee of Maastricht University Medical Center (NL52768.068.15/METC). All procedures were performed at the Maastricht University Medical Center. All subjects provided written informed consent.

## Author Contributions

BD: author and research conductor. FL: co-author and research conductor. NG, AP, JG, and HK: checking the writing and research. MP: co-author and checking the writing and research. RvdB: co-author, conducting the research, and checking the process.

### Conflict of Interest Statement

The authors declare that the research was conducted in the absence of any commercial or financial relationships that could be construed as a potential conflict of interest.
